# Knowledge and Attitude Towards Palliative Care Among Medical Interns in a Tertiary Care Centre in Central India

**DOI:** 10.7759/cureus.104896

**Published:** 2026-03-09

**Authors:** Vikas Pandey, Sumit Kumar, Kalpana Arya, Bikramjeet Mitra

**Affiliations:** 1 Community Medicine, LN Medical College and Research Center, Bhopal, IND; 2 Physiology, Government Medical College, Datia, Datia, IND; 3 Community Medicine, Government Medical College, Datia, Datia, IND

**Keywords:** attitude, central india region, knowledge, medical interns, palliative care

## Abstract

Background

Availability of palliative care services across India has significantly improved, but adequate training remains lacking, largely due to inadequate resources and limited sensitisation, leading to regional and state-wise disparities.

Objectives

The objectives of this study are to evaluate the knowledge and attitudes regarding palliative care among medical interns and to identify the factors influencing their understanding and perspectives on this important aspect of healthcare.

Materials and methods

An observational cross-sectional study was conducted among 112 medical interns at Government Medical College, Datia, Madhya Pradesh, India. Data were collected using a pre-validated structured questionnaire after obtaining written informed consent from the participants. The questionnaire included items on knowledge, attitudes, and factors related to palliative care. In addition, sociodemographic details, including age, sex, education, and residence, were gathered. Statistical analysis was performed using Jamovi (version 2.3.28), and a p-value <0.05 was considered statistically significant.

Results

The study recruited 112 medical interns with a mean age of 24.0 ± 1.49 years. Among the participants, 79 (70.5%) were males. While 102 (91%) interns reported awareness about palliative medicine, 50 (44.6%) had an excellent grade of knowledge regarding palliative care, and 97 (86.6%) medical interns had a positive attitude towards palliative care. There was a significant association between knowledge and attitude levels regarding palliative care (p-value = 0.0022), and notably, 99 (88.4%) medical interns expressed a need to incorporate formal palliative care training into their internship program.

Conclusion

Although nearly half of the interns demonstrated excellent knowledge, a substantial proportion exhibited only fair or moderate understanding, indicating variability in competency levels. There was a significant association between knowledge and attitude levels regarding palliative care among medical interns, and the vast majority acknowledged the need for formal palliative care training during internship to enhance their professional skills and improve patient care outcomes.

## Introduction

According to the World Health Organisation (WHO), palliative care is an "approach that improves the quality of life of patients and their families facing the problems associated with life-threatening illness, through the prevention and relief of suffering by means of early identification and impeccable assessment and treatment of pain and other problems, physical, psychological, and spiritual” [1]. With the global rise in chronic diseases, such as cancer and cardiovascular disorders, the need for palliative care has significantly increased [2]. The goal of palliative care is to enhance the health-related quality of life (HRQOL) of people with life-threatening illnesses, and its multidisciplinary approach focuses on both the patient and family [2]. In the literature on palliative oncology, terminology such as supporting care, optimum supportive care, palliative care, and hospice care is rarely and inconsistently defined [3]. Palliative care can play a significant role in providing relief for both physical and psychological symptoms of the illness in non-communicable diseases (NCDs), such as cancer, diabetes mellitus, cardiovascular disease, and chronic respiratory problems, which account for 65% of all reported deaths globally; however, worldwide, only approximately 14% of people who need palliative care currently receive it [4]. Each year, an estimated 56.8 million people worldwide require palliative care, most of whom live in low- and middle-income countries. For children, 98% of those needing palliative care live in low- and middle-income countries, with most of them living in countries in the Global South [1,4]. It is estimated that 48 million individuals will die annually owing to severe health conditions by 2060 [5]. Palliative care spans the period from the development and progression of the chronic illness through the last phases of the condition and requires a coordinated, interdisciplinary approach that focuses on the patient’s comfort [6].

In the Indian context, the idea of palliative care was introduced in the middle of the 1980s. Since then, hospice and palliative care services have grown, thanks to the dedication of many people, including volunteers and dedicated individuals from India and other nations, as well as international organisations [7]. In the late 1980s and early 1990s, facilities for providing palliative care within cancer centres were first established in cities such as Ahmedabad, Bangalore, Mumbai, Trivandrum, and Delhi [8]. Kerala was the first state in India to formally announce a policy on palliative care, and the Calicut model has also been designated as a WHO demonstration project as a case study for the provision of high-quality, adaptable, and affordable palliative care in the developing world and for exemplifying sound guidelines for collaboration between the government and non-government organisations (NGO) [9]. The National Programme for Palliative Care (2017) has been implemented as part of the National Health Mission, but policies on palliative care have been framed by only four states: Kerala, Maharashtra, Karnataka, and Tamil Nadu [10]. While significant improvements in the availability of palliative care services across India have been observed, regional and state-wise disparities remain high. Only a few state hospitals and cancer centres in India offer palliative care, and corporate hospitals have not yet adopted home care.

The National Medical Commission (NMC), formerly known as the Medical Council of India (MCI), has incorporated palliative care as a core competency in its current competency-based undergraduate curriculum and has initiated a postgraduate program in palliative medicine; however, proper training is still lacking, mostly due to inadequate resources and less sensitisation [11].

Despite increasing recognition of palliative care as an essential component of comprehensive healthcare, structured undergraduate exposure remains limited in many Indian medical institutions. There is limited evidence assessing the preparedness of MBBS interns, who represent the immediate future frontline physicians, in delivering basic palliative care services. In view of the above findings, this study was planned to assess the knowledge and attitude of medical interns at Government Medical College, Datia, about palliative care. This study aimed to assess the knowledge and attitudes of medical interns toward palliative care and analyse the association between their level of knowledge and attitude toward palliative care at Government Medical College, Datia, Madhya Pradesh, India.

## Materials and methods

Study design

This cross-sectional observational study assessed the knowledge and attitudes toward palliative care among medical interns.

Study setting

The study was conducted at the Government Medical College, Datia, Madhya Pradesh, over a six-month period from December 2024 to May 2025.

Study participants

The study population comprised MBBS interns aged 22 years and above, who were undergoing compulsory rotatory internship during the study period, representing all clinical departments and socioeconomic backgrounds. Interns who were present during the study period and willing to participate were enrolled.

Inclusion and exclusion criteria

MBBS interns aged 22 years and above, who were undergoing compulsory rotatory internship during the study period, were included in the study. Interns who declined to participate or submitted incomplete or inadequately filled questionnaires were excluded from the analysis.

Sample size and sampling technique

Based on the currently recorded annual enrolment of MBBS interns (n = 120), a minimum sample size of 92 participants was estimated using Raosoft’s online sample size calculator, assuming a 95% confidence level and 5% margin of error [12]. To improve the precision and robustness of the findings, all eligible and consenting interns available during the study period were approached, and 112 participants were ultimately included.

Study variables

The primary outcome variables were knowledge and attitudes toward palliative care. The independent variables comprised socio-demographic characteristics and factors influencing awareness and perspectives on palliative care.

Data collection tool and measurement

Data were collected using a closed-ended, semi-structured, self-administered questionnaire designed to assess the knowledge, attitudes, and related domains of palliative care (Appendix). Responses were recorded directly from the participants during data collection.

Justification of scoring

The questionnaire consisted of 25 items that assessed knowledge of and attitudes toward palliative care. Knowledge-based questions included items Q1-6, Q10-18, and Q20-21 (total = 16 items), and attitude-based questions included items Q7-9, Q19, and Q22-25 (total = 9 items). Each item was scored dichotomously, with one point awarded for correct or appropriate responses and zero points for incorrect or inappropriate responses. Negatively worded items were reverse-scored prior to the analysis.

Composite scores were calculated separately for the knowledge and attitude domains by summing individual item scores. Knowledge scores were categorised as poor (<50%), fair (50-74%), good (75-89%), or excellent (90-100). Attitude scores were categorised as negative (<50%), neutral (50-74%), or positive (75-100%). Continuous data are presented as mean ± standard deviation.

Bias control

To minimise information bias, the questionnaire was standardised and uniformly administered to all participants. Pre-testing the instrument helped reduce ambiguity and misinterpretation of questionnaire items.

Validity and reliability of the study tool

The content validity of the questionnaire was ensured through expert review by faculty members from the Department of Community Medicine and the Medical Education Unit (MEU) at Government Medical College, Datia. The questionnaire underwent content validation by three subject experts in Community Medicine and Palliative Care. The questionnaire was pretested and revised accordingly. A pilot study was conducted among 10 interns (excluded from final analysis) to assess clarity and feasibility. Internal consistency reliability was assessed using Cronbach’s alpha, which was 0.78, indicating acceptable reliability.

Ethical considerations

Ethical approval was obtained from the Institutional Ethics Committee (IEC), Government Medical College, Datia, vide Project No. 99/CM/GMC/IECBMHR/2024. Written informed consent was obtained from all participants prior to enrolment. The confidentiality and anonymity of the participants’ information were strictly maintained throughout the study.

Statistical analysis

Data were entered into Microsoft Excel (Microsoft Corp., USA) and analysed using Jamovi statistical software (version 2.3.28, The Jamovi Project, retrieved from https://www.jamovi.org, Sydney, Australia). Categorical variables were summarised using frequency and percentage. The Chi-square test was used to assess associations between categorical variables, and a p-value <0.05 was considered statistically significant.

## Results

Among the 112 medical interns included in the study, 79 (70.5%) were male, and 33 (29.5%) were female. The participants' ages ranged from 22 to 28 years, with an average of 24.01 ± 1.49 years. Most interns, 71 (63.4%), were in the 22-24 years age group (Table 1).

**Table 1:** Sociodemographic characteristics, knowledge, and attitude regarding palliative care among the medical interns Data are expressed as frequency and percentage. Age is presented in completed years. Knowledge and attitude levels were categorized based on predefined composite scoring criteria.

Parameters	Distribution	Frequency; n (%)
Age (In years)	22–24	71 (63.4%)
	25–28	41 (36.61%)
Gender	Male	79 (70.5%)
	Female	33 (29.5%)
Knowledge level	Poor	08 (7.10%)
	Fair	28 (25.00%)
	Good	26 (23.20%)
	Excellent	50 (44.60%)
Attitude level	Negative	06 (5.40%)
	Neutral	09 (8.00%)
	Positive	97 (86.60%)

Awareness of palliative medicine was reported by 102 (91.0%) interns, and 71 (63.0%) had prior experience in this field. In addition, 74 (66.0%) participants reported caring for a dying family member, and 78 (70.0%) were aware of the concept of bereavement. However, only 67 (60.0%) interns correctly identified the appropriate time to initiate palliative care (Table 2). A large majority, 105 (94.0%), agreed that palliative care can be provided alongside curative treatment (Table 2).

**Table 2:** Item-wise distribution of responses to the palliative care knowledge and attitude questionnaire among medical interns (n = 112) WHO: World Health Organization, DNAR/DNR: do not attempt resuscitation/do not resuscitate. Data expressed as frequency and percentage.

Question no.	Questionnaire item	Yes n (%)	No n (%)
Awareness and concepts			
Q1	Are you aware of palliative medicine?	102 (91.0)	10 (9.0)
Q2	Do you have any experience with palliative care?	71 (63.0)	41 (37.0)
Q3	Have you experienced caring for dying family members?	74 (66.0)	38 (34.0)
Q4	Do you think palliative care is needed for total care (physical, psychological, social, spiritual)?	67 (60.0)	45 (40.0)
Q5	Do you know what bereavement is?	78 (70.0)	34 (30.0)
Q6	Are you aware of the concept of bereavement care?	73 (65.0)	39 (35.0)
Attitude toward care and communication			
Q7	Taking care of the caregiver is as important as patient care	107 (95.5)	5 (4.5)
Q8	Prognosis should always be clearly communicated to the patient	103 (92.0)	9 (8.0)
Philosophy of palliative medicine			
Q9	The provision of palliative care requires emotional detachment	76 (67.9)	36 (32.1)
Q10	Palliative care should only be provided to patients with no curative options	68 (60.7)	44 (39.3)
Q11	Palliative care can be provided alongside curative treatment	105 (93.8)	7 (6.2)
Pain management			
Q12	Do you know about the WHO pain scale?	84 (75.0)	28 (25.0)
Q13	Is pain considered a vital sign?	99 (88.4)	13 (11.6)
Q14	Should the severity of pain determine the method of treatment?	103 (92.0)	9 (8.0)
Q15	Is morphine the most effective drug for cancer pain?	93 (83.0)	19 (17.0)
Q16	Does morphine use in palliative care cause addiction?	86 (76.8)	26 (23.2)
Dyspnoea management			
Q17	Can morphine be used to manage dyspnoea in palliative care patients?	72 (64.3)	38 (35.7)
Q18	Can oxygen supplementation help during terminal dyspnoea?	99 (88.4)	13 (11.6)
Q19	Resuscitation must always be performed on crashing patients	96 (85.7)	16 (14.3)
Gastrointestinal and nursing issues			
Q20	Are you aware of the care and challenges related to colostomy patients?	90 (80.4)	22 (19.6)
Q21	Is central venous access the only option if peripheral IV access is not possible?	92 (82.1)	20 (17.9)
Q22	Should patients and relatives both be involved in DNAR/DNR decisions?	99 (88.4)	13 (11.6)
Q23	Should nurses address only the physical aspects of disease?	86 (76.8)	26 (23.2)
Q24	Should family members be involved in the physical care of the dying patient?	102 (91.1)	10 (8.9)
Q25	Is formal palliative care training necessary for medical interns?	105 (93.8)	7 (6.2)

Overall, 44.6% of the participants demonstrated excellent knowledge, followed by fair (25.0%), good (23.2%), and poor (7.0%) knowledge. Among males (n = 79), the highest proportion exhibited excellent knowledge (78.0%), whereas among females (n = 33), knowledge distribution was more varied, with 42.31% showing good and 28.57% fair knowledge. However, the association between gender and knowledge level was not statistically significant (χ² = 3.66, p = 0.30). Similarly, most interns aged 18-24 years (60.0%) and 25-30 years (40.0%) demonstrated excellent knowledge, with no significant association observed between age group and knowledge category (χ² = 1.38, p = 0.71) (Table 3).

**Table 3:** Association of the sociodemographic characteristics with the distribution of knowledge levels of palliative care among medical interns This table presents the relationship between gender and age categories with levels of knowledge (poor, fair, good, and excellent) about palliative care among 112 medical interns. Knowledge scores were categorized as poor for scores below 50% (<8 points), fair for scores between 50% and 74% (8–11 points), good for scores between 75% and 89% (12–14 points), and excellent for scores of 90% or higher (15–16 points). Data are expressed as frequency with row percentages in parentheses. Associations were tested using the Chi-square test. NS: not significant (p > 0.05)

Parameters	Response options	Poor n = 8 (7%)	Fair n=28 (25%)	Good n = 26 (23.2%)	Excellent n = 50 (44.6%)	Total n = 112 (100%)	Chi-square	p-value
Gender	Male	5 (62.5)	20 (71.43)	15 (57.69)	39 (78)	79 (70.54)	3.66	0.3 (NS)
									Female	3 (37.5)	8 (28.57)	11 (42.31)	11 (22)	33 (29.46)
	Age	22–24	5 (62.5)	17 (60.71)	19 (73.08)	30 (60)			71 (63.39)	1.38	0.71 (NS)
25–28							3 (37.5)	11 (39.29)				7 (26.92)	20 (40)	41 (36.61)

A predominantly positive attitude was observed in 86.6% of interns, while neutral and negative attitudes were reported in 8.0% and 5.4%, respectively. Among males, 71.13% exhibited a positive attitude, compared to 28.87% of females. Although a greater proportion of older interns (25-30 years) demonstrated positive attitudes (39.18%) with no neutral responses in this subgroup, neither gender (χ² = 1.46, p = 0.48) nor age (χ² = 5.93, p = 0.06) showed a statistically significant association with attitude levels (Table 4).

**Table 4:** Association of the socio-demographic characteristics with the distribution of attitude levels of palliative care among medical interns This table shows the association between gender and age groups with attitude categories (negative, neutral, and positive) toward palliative care among the study participants. Attitude scores were categorized as negative for scores below 50% (<5 points), neutral for scores between 50% and 74% (5–6 points), and positive for scores of 75% or higher (7–9 points). Values are presented as frequency with row percentages in parentheses. Chi-square test was used as a test of association. NS: not significant (p > 0.05)

Parameters	Response options	Negative n = 6 (5.4%)	Neutral n = 9 (8.0%)	Positive, n = 97 (86.60%)	Total n = 112 (100%)	Chi-square (x2)	p-value
								Gender	Male	5 (83.33)	5 (55.56)	69 (71.13)	79 (70.54)	1.46	0.48 (NS)
																Female	1 (16.67)	4 (44.44)	28 (28.87)	33 (29.46)
Age	22– 24	3 (50)	9 (100)	59 (60.82)	71 (63.39)	5.93	0.06 (NS)								
	25– 28	3 (50)	0 (0)	38 (39.18)	41 (36.61)		

Interns with excellent knowledge uniformly demonstrated positive attitudes (100%). By contrast, negative and neutral attitudes were more frequent among those with poor or fair knowledge (χ² = 20.585, p = 0.0022). Specifically, 50% of participants with poor knowledge exhibited negative attitudes, and 66.67% of those with fair knowledge demonstrated neutral attitudes (Table 5).

**Table 5:** Association between the knowledge and attitude levels regarding palliative care among medical interns This table depicts the cross-tabulation between knowledge levels (poor, fair, good, excellent) and attitude levels (negative, neutral, positive) toward palliative care. Frequencies with percentages are shown. Chi-square test was used as test of association. S: statistically significant (p < 0.05)

Parameters	Response options	Attitude			Total n = 112 (%)	Chi-square	p-value
		Negative, n = 6 (%))	Neutral, n=9 (%)	Positive, n = 97 (%)			
Knowledge	Poor	3 (50%)	1 (11.1%)	4 (4.12%)	8 (7.14%)	20.585	0.0022 (S)
	Fair	2 (33.3%)	6 (66.67%)	20 (20.62%)	28 (25%)		
	Good	1 (16.67%)	2 (22.22%)	23 (23.71%)	26 (23.21%)		
	Excellent	0 (0%)	0 (0%)	50 (100%)	50 (44.64%)		

Overall, compared with earlier Indian and international studies, the present findings indicate meaningful progress in awareness, knowledge, and attitudes toward palliative care among medical interns. However, persistent gaps in early initiation and practical application of palliative care highlight the need for structured, competency-based, and practice-oriented training during the internship period to ensure effective translation of knowledge into clinical practice.

## Discussion

The present study evaluated knowledge and attitudes toward palliative care among medical interns in a tertiary care setting. It demonstrated a high level of awareness (91.0%) and a substantial proportion of interns with good-to-excellent knowledge (67.8%). These findings differ from those reported by Divya et al., who observed inadequate knowledge of palliative care among medical interns and noted that undergraduate medical education at that time was largely focused on curative treatment, with minimal emphasis on end-of-life care (EOLC) [13]. The higher knowledge levels observed in the present study suggest a gradual improvement over time, likely reflecting increased exposure to palliative care concepts during undergraduate medical training.

Suresh et al. reported low mean knowledge scores among interns (3.44 ± 1.18) and identified a general deficiency in understanding EOLC among medical undergraduates, with inconsistent knowledge levels across training stages [14]. By contrast, nearly half of the interns in the present study (44.6%) achieved an excellent knowledge score, indicating a more consistent and improved baseline understanding. This improvement may be related to curricular reforms and greater institutional emphasis on palliative care in recent years.

A key finding of the present study is that 94.0% of interns recognized that palliative care can be provided alongside curative treatment. Earlier studies frequently reported misconceptions, with palliative care perceived as appropriate only when curative options were exhausted [13,14]. Evidence from educational interventional studies supports the effectiveness of structured teaching in addressing such misconceptions. An Indian interventional study demonstrated a significant improvement in interns’ knowledge following targeted palliative care training, with mean scores increasing from 8.82 ± 2.13 to 14.44 ± 1.72 (p < 0.0001) [15]. Similarly, a quasi-experimental study in Nigeria found significant improvement in interns’ knowledge following a structured educational intervention [16]. The variability in knowledge levels may be attributed to limited structured exposure to palliative care during undergraduate training, inconsistent clinical postings in oncology or hospice settings, and a lack of formal competency-based modules. Additionally, cultural perceptions regarding end-of-life care may influence learning priorities.

In the present study, awareness of bereavement (70.0%) and recognition that pain management should be guided by disease severity (92.0%) were relatively high. However, only 60.0% of interns correctly identified the appropriate timing for initiating palliative care, indicating a gap between theoretical knowledge and clinical application. This finding is consistent with international evidence. A mixed-methods study among nursing interns in China reported moderate knowledge levels despite positive attitudes and identified the absence of standardized education as a major barrier to applying knowledge in practice [17]. Similarly, a systematic review and meta-analysis from Ethiopia reported a low pooled prevalence of palliative care knowledge among nurses (42.3%), with education and prior training emerging as key determinants of knowledge [18].

The present study also demonstrated a statistically significant association between knowledge and attitudes toward palliative care, indicating that higher levels of knowledge are associated with more favourable professional attitudes. This contrasts with the findings of Suresh et al., where 33.4% of respondents did not perceive the need for EOLC training, reflecting less favourable attitudes [14]. In comparison, 86.6% of interns in the present study exhibited a positive attitude toward palliative care, suggesting a notable improvement. This pattern is consistent with evidence indicating that education and clinical exposure play a critical role in shaping attitudes toward palliative and end-of-life care [15-17].

Importantly, 88.4% of interns in the present study expressed the need for formal palliative care training during the internship period. This aligns with findings from a Delhi-based study among critical care physicians, where lack of training was identified as a major barrier to effective EOLC, and there was strong support for curriculum-based education [19]. These observations are further supported by national policy and expert consensus recommendations advocating the mandatory integration of palliative care education at undergraduate and postgraduate levels [20].

Overall, compared with earlier Indian and international studies, the present findings indicate meaningful progress in awareness, knowledge, and attitudes toward palliative care among medical interns. However, persistent gaps in early initiation and practical application of palliative care highlight the need for structured, competency-based, and practice-oriented training during the internship period to ensure effective translation of knowledge into clinical practice.

Limitation

As it is a single-centre study, the findings may not be generalizable to other medical institutions. The sample size was limited to the number of available interns during the study period. As data were collected using a self-administered questionnaire, responses may be subject to social desirability and recall bias. Additionally, the cross-sectional design limits causal inference regarding factors influencing knowledge levels. These findings underscore the need to integrate structured palliative care modules into the undergraduate curriculum, including competency-based training, simulation exercises, and supervised clinical exposure. Strengthening early exposure could improve preparedness and ensure holistic delivery of patient care.

## Conclusions

Many interns demonstrated a good-to-excellent understanding of palliative care; however, there remains significant variability in their knowledge levels. This highlights the need for structured, competency-based palliative care training within undergraduate medical education. By integrating formal curriculum modules, providing dedicated clinical placements, and implementing regular assessments, we can better prepare future physicians to deliver comprehensive end-of-life care.

## Appendices

**Table 6:** Structured questionnaire for the assessment of knowledge and attitude toward palliative care among medical interns, including item-wise domains and scoring framework Calculation of the knowledge score Knowledge regarding palliative care was assessed using 16 items that evaluated awareness, conceptual understanding, and clinical application of palliative care principles. These items encompassed the introduction to palliative care (items B1–B6), philosophy of palliative medicine (items C2 and C3), pain management (items D1–D5), dyspnoea management (items E1 and E2), and gastrointestinal and nursing issues (items F1 and F2). Each correct response was awarded one point, while incorrect responses received zero points, yielding a maximum possible knowledge score of 16. Knowledge scores were categorized as poor for scores below 50% (<8 points), fair for scores between 50% and 74% (8–11 points), good for scores between 75% and 89% (12–14 points), and excellent for scores of 90% or higher (15–16 points). Calculation of the attitude score Attitude toward palliative care was assessed using nine items focusing on ethical orientation, communication, teamwork, and training needs. These included items from the introduction to palliative care section (items B7 and B8), philosophy of palliative medicine (item C1), dyspnoea management (item E3), and gastrointestinal and nursing issues (items F3–F6). Each response reflecting a positive attitude was scored as one point, while negative or incorrect responses were scored as zero, resulting in a maximum attitude score of nine. Attitude scores were categorized as negative for scores below 50% (<5 points), neutral for scores between 50% and 74% (5–6 points), and positive for scores of 75% or higher (7–9 points).

Section	Item	Question / statement	Response format
Section A: Demographic Characteristics	A1	Name	____________________
	A2	Age (in years)	______
	A3	Sex	☐ Male ☐ Female ☐ Other
Section B: Introduction to Palliative Care	B1	Are you aware of Palliative Medicine?	☐ Yes ☐ No
	B2	Do you have any experience of Palliative Care?	☐ Yes ☐ No
	B3	Have you experienced caring for dying family members?	☐ Yes ☐ No
	B4	When do you think palliative care is needed?	☐ Care of terminally ill patients ☐ Total care (physical, psychological, social, spiritual) ☐ HIV/AIDS and chronic illnesses ☐ All of the above
	B5	Do you know what bereavement is?	☐ Yes ☐ No
	B6	Are you aware of the concept of bereavement care?	☐ Yes ☐ No
	B7	Taking care of the caregiver is equally important as patient’s care.	☐ Yes ☐ No
	B8	Prognosis should always be clearly communicated to the patient.	☐ Yes ☐ No
Section C: Philosophy of Palliative Medicine	C1	The provision of palliative care requires emotional detachment from the healthcare professional.	☐ Yes ☐ No
	C2	Palliative care should only be provided to patients with no curative treatment options.	☐ True ☐ False
	C3	Palliative care can be provided alongside curative treatment.	☐ True ☐ False
Section D: Pain Management	D1	Do you know about the WHO Pain Scale?	☐ Yes ☐ No
	D2	Is pain considered a vital sign?	☐ Yes ☐ No
	D3	The severity of pain should determine the method of treatment.	☐ Yes ☐ No
	D4	Is morphine the most effective drug for cancer pain?	☐ Yes ☐ No
	D5	Does morphine in palliative care cause addiction in terminally ill patients?	☐ Yes ☐ No
Section E: Dyspnoea Management	E1	Morphine can be used to manage dyspnoea in palliative care patients.	☐ Yes ☐ No
	E2	Oxygen supplementation may help during the last difficult breaths.	☐ Yes ☐ No
	E3	Resuscitation must always be performed on crashing patients, irrespective of advanced metastatic cancer.	☐ Yes ☐ No
Section F: Gastrointestinal and Nursing Issues	F1	Are you aware of the care and challenges related to a patient with a colostomy?	☐ Yes ☐ No
	F2	There is no route except central venous access for patients unable to maintain peripheral intravenous access.	☐ Yes ☐ No
	F3	Patients and relatives should both be involved in DNAR/DNR decision-making if possible.	☐ Yes ☐ No
	F4	Nurses should address only the physical aspects of disease; psychological issues must be handled by psychiatrists or other professionals.	☐ Yes ☐ No
	F5	The family should be involved in the physical care of the dying person.	☐ Yes ☐ No
	F6	Do you think formal palliative care training is necessary for medical interns?	☐ Yes ☐ No
